# A Side by Side Comparison of Bruker Biotyper and VITEK MS: Utility of MALDI-TOF MS Technology for Microorganism Identification in a Public Health Reference Laboratory

**DOI:** 10.1371/journal.pone.0144878

**Published:** 2015-12-10

**Authors:** Simon Lévesque, Philippe J. Dufresne, Hafid Soualhine, Marc-Christian Domingo, Sadjia Bekal, Brigitte Lefebvre, Cécile Tremblay

**Affiliations:** 1 Laboratoire de santé publique du Québec / Institut national de santé publique du Québec, Sainte-Anne-de-Bellevue, Québec, Canada; 2 Département de Microbiologie, Immunologie et Infectiologie, Université de Montréal, Montréal, Québec, Canada; 3 Centre de Recherche du Centre Hospitalier de l’Université de Montréal, Montréal, Québec, Canada; California Department of Public Health, UNITED STATES

## Abstract

Matrix-assisted laser desorption ionization–time of flight mass spectrometry (MALDI-TOF MS) has emerged as a rapid, highly accurate, and cost-effective method for routine identification of a wide range of microorganisms. We carried out a side by side comparative evaluation of the performance of Bruker Biotyper versus VITEK MS for identification of a large and diverse collection of microorganisms. Most difficult and/or unusual microorganisms, as well as commonly encountered microorganisms were selected, including Gram-positive and negative bacteria, mycobacteria, actinomycetes, yeasts and filamentous fungi. Six hundred forty two strains representing 159 genera and 441 species from clinical specimens previously identified at the Laboratoire de santé publique du Québec (LSPQ) by reference methods were retrospectively chosen for the study. They included 254 Gram-positive bacteria, 167 Gram-negative bacteria, 109 mycobacteria and aerobic actinomycetes and 112 yeasts and moulds. MALDI-TOF MS analyses were performed on both systems according to the manufacturer’s instructions. Of the 642 strains tested, the name of the genus and / or species of 572 strains were referenced in the Bruker database while 406 were present in the VITEK MS IVD database. The Biotyper correctly identified 494 (86.4%) of the strains, while the VITEK MS correctly identified 362 (92.3%) of the strains (excluding 14 mycobacteria that were not tested). Of the 70 strains not present in the Bruker database at the species level, the Biotyper correctly identified 10 (14.3%) to the genus level and 2 (2.9%) to the complex/group level. For 52 (74.2%) strains, we obtained no identification, and an incorrect identification was given for 6 (8.6%) strains. Of the 178 strains not present in the VITEK MS IVD database at the species level (excluding 71 untested mycobacteria and actinomycetes), the VITEK MS correctly identified 12 (6.8%) of the strains each to the genus and to the complex/group level. For 97 (54.5%) strains, no identification was given and for 69 (38.7%) strains, an incorrect identification was obtained. Our study demonstrates that both systems gave a high level (above 85%) of correct identification for a wide range of microorganisms. However, VITEK MS gave more misidentification when the microorganism analysed was not present in the database, compared to Bruker Biotyper. This should be taken into account when this technology is used alone for microorganism identification in a public health laboratory, where isolates received are often difficult to identify and/or unusual microorganisms.

## Introduction

Identification of microorganisms in a clinical microbiology laboratory provides definitive knowledge about the cause of infection and plays a critical role in patient management and choice of antimicrobial treatment. Traditionally, the identification of bacteria and fungi in clinical laboratories has relied on phenotypic methods, such as growth on selective and non-selective media, morphology of colonies, Gram-stain, microscopic morphology and typical biochemical reactions, using multiple automated and/or manual testing methods [1]. These methods are laborious, time consuming and require a relatively long turn-around time. In addition, unusual and difficult-to-identify organisms encountered in the clinical laboratory may need to be sent to a reference laboratory for ultimate identification, often obtained by DNA sequencing.

Matrix-assisted laser desorption ionization–time of flight mass spectrometry (MALDI-TOF MS) has emerged as a rapid, highly accurate, and cost-effective method for routine identification of a wide range of microorganisms [2,3]. The technology is now widespread, as readily evidenced by the ever increasing number of publications describing the use of this method for identifying clinical isolates of microorganisms [4]. In North America, there are two commercially available MALDI-TOF MS systems for microbial identification, the Bruker Biotyper (Bruker Daltonics) and the VITEK MS^TM^ PLUS (bioMérieux) (hereinafter named VITEK MS). A small number of studies have simultaneously evaluated the performance of multiple MALDI-TOF MS systems and found them comparable for the identification of a variety of aerobic and anaerobic bacteria and yeast [5].

The majority of MALDI-TOF MS evaluation study designs target the identification of common microorganisms routinely identified in clinical laboratories. To our knowledge, only one study has evaluated the accuracy of both VITEK MS and Bruker Biotyper for the identification of the most difficult and/or unusual microorganisms [6]. The authors found no significant difference in the number of organisms identified to the genus level, species level, unidentified or misidentified by the two MALDI-TOF MS systems. Despite having evaluated an extensive clinical collection of microorganisms the most difficult and/or unusual microorganisms, the vast majority of genus and species were present in both software databases, except for filamentous fungi.

In the current study, we carried out a side by side comparative evaluation of the performance of Bruker Biotyper versus VITEK MS for identification of a large and diverse collection of microorganisms. Most difficult to identify and/or unusual microorganisms, as well as commonly encountered microorganisms were selected, including Gram-positive and -negative bacteria, mycobacteria, actinomycetes, yeasts and filamentous fungi. The first objective of our study was to evaluate the performance of microorganism identification of both systems in comparison with methods used in a Public Health Reference Laboratory. The second objective was to evaluate the response of both systems for the identification of microorganisms which are not present in the software database.

## Materials and Methods

### Source and Type of Microorganisms Tested

The Laboratoire de santé publique du Québec (LSPQ) is the reference microbiology laboratory for the identification of microorganisms received from clinical laboratories in the province of Quebec. Microorganism identification is part of the mandate of the LSPQ, so no ethical approval was needed for this study. Six hundred forty two strains representing 159 genera and 441 species from clinical specimens previously identified at the LSPQ were retrospectively chosen for the study. They include 254 Gram-positive bacteria, 167 Gram-negative bacteria, 109 mycobacteria and aerobic actinomycetes and 112 yeasts and mould. All microorganisms were previously identified according to LSPQ reference methods: 16S rRNA [7] or *tuf* gene sequencing for Gram-positive bacteria, 16S rRNA, *rpoB* [8] or *cpn60* gene [9] sequencing for Gram-negative bacteria, conventional biochemical testing for *Enterobacteriaceae* [10], 16S rRNA or *hsp*65 gene sequencing [11] for mycobacteria and actinomycetes and ribosomal DNA (ITS / D1D2 regions) [12,13] or beta tubulin gene (*benA*) [14,15] sequencing for yeast and fungi. All microorganisms were identified at the LSPQ from 2009 to 2013. Strains were stored at -80°C and subcultured on the appropriate culture media to obtain sufficient growth.

### MALDI-TOF MS Identification with Bruker Biotyper

Depending on the microorganism, different identification protocols were used according to the manufacturer’s instructions. For bacteria and yeasts up to three different extraction procedures were used. The “direct colony extraction technique” by which the microbes are directly applied as a thin film on a spot of the target slide (a reusable 96 wells stainless steel target slide, Bruker Daltonics), allowed to dry at room temperature, followed by the addition of 1 μl of HCCA matrix onto each sample spot dried at room temperature prior to MS analysis. The “on slide formic acid extraction” technique consisted of the addition of 1 μl of 70% formic acid directly onto the dried bacterial or yeast spot that was dried once more prior to adding the HCCA matrix.

The “in tube formic acid extraction” technique was performed by adding one or two colonies to a microcentrifuge tube containing 300 μl of HPLC grade deionized water. The suspension was mixed thoroughly by pipetting, and 900 μl of absolute ethanol was further added and mixed thoroughly again. After a centrifugation of 2 minutes at 13 000 rpm, the supernatant was discarded and 50 μl of 70% formic acid was added to the pellet and vortexed. Fifty μl of 100% acetonitrile was added and mixed, followed by a 2 minute centrifugation at 13 000 rpm. One μl of the supernatant was added to the target slide and dried at room temperature. One μl of the HCCA matrix was applied on the sample spot and dried at room temperature.

For mycobacteria and aerobic actinomycetes, the inactivation extraction method was performed as follows. A loopful of microorganisms from a solid culture medium (Middelbrook 7H10 for mycobacteria and Sabouraud agar for aerobic actinomycetes) was added to 300 μl of HPLC grade deionized water into an Eppendorf tube. The solution was inactivated by heat for 30 minutes at 100°C. After a centrifugation of 2 minutes at 13 000 rpm, the supernatant was discarded and 300 μl of HPLC grade deionized water was added. Then, 900 μl of absolute ethanol was added and vortexed thoroughly. After centrifugation of 2 minutes at 13 000 rpm, the supernatant was discarded and 500 μl of HPLC grade deionized water was added and vortexed in order to completely suspend the pellet. After centrifugation of 2 minutes at 13 000 rpm, the supernatant was discarded and 50 μl of HPLC grade deionized water was added and vortexed. The solution was heated for 10 minutes at 95°C. Once the solution had cooled down, 1200 μl of pre-cooled (-20°C) absolute ethanol was added. Since the previous steps were performed in a containment level 3 laboratory, viability tests for the *Mycobacterium tuberculosis* (MTB) complex isolates were assessed by plating the crude extract to ensure the cells had all been inactivated before proceeding to MS identification. The extracts were stored at (-20°C) during 6 weeks required for the completion of the viability tests. Once confirmed as inactivated, the extracts were centrifuged for 2 minutes at 13 000 rpm, the supernatant was discarded and the pellet was completely air dried. Fifty μl of 100% acetonitrile and 0.5 mm Zirconia / Silica beads (BioSpec Products) were added and vortexed at maximum speed for 1 minute. The extract was supplemented with 50 μl of 70% formic acid and vortexed for 5 seconds. The suspension was centrifuged for 2 minutes at 13 000 rpm. One μl of the supernatant was added to the target slide and dried at room temperature. One μl of the HCCA matrix was applied on the sample spot and dried at room temperature.

For moulds, the cultivation procedure was performed as follows. From sufficient growth on a Sabouraud agar plate, a Sabouraud liquid media tube was inoculated with a few well isolated colonies and closed tightly. Tubes were placed into a rotator to shake over head. After 24–48h of growth in the rotator, the tube was placed in a standing rack for 10 minutes to let the fungal culture settle at the bottom of the tube. Then, 1.5 ml of sedimented fraction was harvested and transferred into an Eppendorf tube. The tube was centrifuged for 2 minutes at 13 000 rpm and the supernatant discarded. One ml of HPLC grade deionized water was added to the pellet and vortexed for one minute. The tube was centrifuged for 2 minutes at 13 000 rpm and the supernatant was discarded. The pellet was washed one more time with water. The pellet was suspended in 300 μl of HPLC grade deionized water and 900 μl of absolute ethanol, and vortexed. The tube was centrifuged for 2 minutes at 13 000 rpm, the supernatant was discarded and the pellet was allowed to completely dry. Seventy percent formic acid was added proportionally to the size of the pellet. The same amount of 100% acetonitrile was added and mixed. The tube was centrifuged for 2 minutes at 13 000 rpm. One μl of the supernatant was added to the target slide and dried at room temperature. One μl of the HCCA matrix was applied to the sample spot and dried at room temperature.

Each microorganism tested was spotted twice on the same target slide. Measurement was performed on a MALDI Biotyper using the manufacturer's suggested settings using automated collecting spectra. Captured spectra were analyzed using MALDI Biotyper automation control and Bruker Biotyper 3.1 software containing the library number 3995. For each run, a standard (bacterial test standard, Bruker Daltonics) was included to calibrate the instrument and validate the run. Identification criteria were used as recommended by the manufacturer: a score of ≥2.000 indicated species level identification, a score between 1.700 and 1.999 indicated identification to the genus level and a score <1.700 was interpreted as no identification. For bacteria, if a good identification was not obtained with the direct colony technique, subsequent deposit was done with the on slide formic acid extraction technique, followed by the in tube formic acid extraction technique.

### MALDI-TOF MS Identification with bioMérieux VITEK MS

According to the manufacturer's instructions, different protocols were used depending on the microorganism. For bacteria and yeast, the direct colony technique consisted of applying the microbe as a thin film on a spot of the target slide (disposable 48 wells target slide, bioMérieux) and allowed to visibly dried at room temperature. Subsequently, 1 μl of CHCA matrix was applied on each sample spot and dry at room temperature. The on slide formic acid extraction technique consisted of adding 0.5 μl of 25% formic acid on the bacterial or yeast spot and allowing it to completely dry before the addition of the CHCA matrix.

For mycobacteria and actinomycetes, the inactivated method was performed as follows. Two-thirds of a 10 μl calibrated loop of microorganisms from a solid culture media was added to 50 μl of trifluoroacetic acid (Sigma-Aldrich) and vortexed. After 30 minutes at room temperature, 450 μl of HPLC grade deionized water was added to the tube and vortexed. Since the previous steps were performed in a containment level 3 laboratory for the MTB complex, viability testing was performed by plating the solution to ensure that all the cells were inactivated before proceeding to identification. During the viability test, the solution was stored at -20°C. Once inactivation was confirmed, the solution was centrifuged for 2 minutes at 10 000 rpm. One μl of the supernatant was added to the target slide and dried at room temperature. One μl of the CHCA matrix was applied on the sample spot and dried at room temperature.

For moulds, the cultivation procedure was performed as follows. From sufficient growth on a Sabouraud dextrose agar plate, a wet swab was used to swab 1 cm^2^ on the periphery of the plate and suspended in API suspension medium. Nine hundred μl of absolute ethanol was added and vortexed. The tube was then centrifuged for 2 minutes at 10 000 rpm and the supernatant was discarded. Forty μl of 70% formic acid was added to the pellet and vortexed. Forty μl 100% acetonitrile was added and vortexed. The tube was centrifuged for 2 minutes at 10 000 rpm. One μl of the supernatant was added to the target slide and dried at room temperature. One μl of the CHCA matrix was applied on the sample spot and dried at room temperature.

Each microorganism tested was deposited twice on the same target slide. Measurement was performed on a VITEK MS using the manufacturer's suggested settings using automated collecting spectra. Captured spectra were analyzed using VITEK MS automation control and Myla software that included IVD database version 2.0. The VITEK MS target slide consists of 48 sample spots divided into three acquisition groups. For each acquisition group, a standard (*E*. *coli* ATCC 8739) was included to calibrate the instrument and validate the run. The identification criteria used were those as recommended by the manufacturer: a score of ≥60% indicated species level identification. Obtaining a single genus, regardless of the confidence value, was considered an acceptable genus level of identification. If a split identification containing multiple genera, identification was considered unacceptable and considered unidentified. If no identification was provided, the isolate was considered unidentified. For bacteria, if a good identification was not obtained with the direct colony technique, subsequent deposit was done with the on slide formic acid extraction technique.

### Settings of the Study and Discrepancy Analysis

Side by side evaluation was performed on site at the LSPQ from February 2013 to April 2013. Standard training on both MS platforms was provided by manufacturers to the technical team. The same culture plates of each microorganism were used for both systems testing. The same technician performed all the manipulation for bacteria, yeasts and moulds, and a different technician for mycobacteria and actinomycetes. Data were analyzed as follows: LSPQ reference identification was considered as the gold standard. If a discrepancy occurred between Biotyper or VITEK MS and LSPQ identification, MALDI-TOF MS identification was repeated. If a discrepancy occurred between both Biotyper and VITEK and LSPQ identification, LSPQ identification was repeated, according to reference methods mentioned above, only if Biotyper and VITEK MS gave the same results. If there was a discrepancy between the results of the repeated spot on the same technology, only the correct identification (if there was one) was considered. Correct identification was accepted to the species, genus or complex/group level. High score identification (≥2.000 or ≥60%) to a species which was different from the LSPQ identification was considered as wrong identification, even if it was the correct genus. Bad or no spectra acquisition still obtained after one repeat was considered as no identification.

### Statistical Analysis

Data were compared by Chi-square test or Bhapkar test [16] with a significance level of *p*<0.05. Statistical analysis was performed using Statistix version 7.1 (Analytical Software, Tallahassee, FL, USA) and SAS version 9.4 (SAS Canada, Toronto, ON).

## Results

### Identification when the Microorganism Is Present in the Databases

Of the 642 strains tested, 572 were referenced in the Bruker database while 406 were referenced in the VITEK MS IVD database. Of this number, 14 mycobacteria strains belonging to the MTB complex could not be tested with VITEK MS as we found that the inactivation protocol provided by bioMérieux at the time of the study was not functional and the strains were still viable. Of the 572 strains present in the Bruker database, the Biotyper correctly identified 494 (86.4%) strains: 371 (75.1%) strains to species level, 102 (20.6%) to the genus level and 21 (4.3%) to the complex/group level. For 57 (10%) strains, no identification was obtained and 21 (3.6%) strains were misidentified (Tables 1 and 2, S1 and S2 Tables). Among these incorrect identifications, 15 failed to species level and 6 to genus level (Table 3). Of the 392 strains present in the VITEK MS IVD database (excluding the 14 mycobacteria that were not tested), the VITEK MS correctly identified 362 (92.3%) strains: 319 (88.1%) strains to species level, 33 (9.1%) to the genus level and 10 (2.8%) to the complex/group level. For 20 (5.1%) strains, no identification was obtained and for 10 (2.6%) strains, an incorrect identification was provided (Tables 1 and 4, S1 and S3 Tables). Among these incorrect identifications, there were 3 incorrect identifications to species level and 7 to genus level (Table 3). No statistically significant differences between the two systems were found upon comparison of the identification performance for the 380 strains present in both databases (Bhapkar value of 1.61, *p* = 0.4470) (Table 1).

**Table 1:** Identification results when the microorganism is present in both databases.

Reference identification	Number of isolates	Bruker Biotyper					VITEK MS (IVD)				
		Correct identification to the level of					Correct identification to the level of				
		Species	Genus	Complex/group	No ID	Mis ID	Species	Genus	Complex/group	No ID	Mis ID
Gram-positive cocci	104	79	7	13	3	2	93	7	2	1	1
Anaerobes	5	5	0	0	0	0	5	0	0	0	0
Other Gram-positive rods	75	53	12	3	5	2	47	19	3	4	2
Enterobacteriaceae	17	13	1	1	1	1	15	1	1	0	0
Non-fermentative Gram-negative rods	42	32	1	2	4	3	32	3	3	2	2
Other Gram-negative bacteria	66	55	6	0	0	5	64	0	0	0	2
Mycobacteriaceae	18	5	13	0	0	0	16	1	0	0	1
Actinomycetes	8	3	2	0	3	0	5	1	0	1	1
Filamentous fungi	17	11	4	0	2	0	7	1	0	8	1
Yeast	28	22	1	0	4	1	26	0	0	2	0
**Total number of strains (%)**	**380**	**278 (73.2)**	**47 (12.4)**	**19 (5)**	**22 (5.8)**	**14 (3.6)**	**310 (81.6)**	**33 (8.7)**	**9 (2.4)**	**18 (4.7)**	**10 (2.6)**

No ID = No identification obtained. Mis ID = Misidentification obtained

**Table 2:** Identification results when the microorganism is present only in the Bruker Biotyper database.

Reference identification	Number of isolates	Bruker Biotyper				
		Correct identification to the level of				
		Species	Genus	Complex/group	No ID	Mis ID
Gram-positive cocci	24	14	7	0	0	3
Anaerobes	1	1	0	0	0	0
Other Gram-positive rods	33	21	7	0	3	2
Non-fermentative Gram-negative rods	13	9	2	0	1	1
Other Gram-negative bacteria	21	19	1	0	0	1
Mycobacteriaceae	47	10	25	2	10	0
Actinomycetes	20	2	6	0	12	0
Filamentous fungi	26	13	5	0	8	0
**Total number of strains (%)**	**192**	**93 (48.4)**	**55 (28.7)**	**2 (1)**	**35 (18.2)**	**7 (3.6)**

No ID = No identification obtained. Mis ID = Misidentification obtained

**Table 3:** Misidentification by both systems when the microorganisms is referenced in the database.

		Reference identification	Proposed ID (confidence value)^a^
**Bruker Biotyper**	**Misidentification to species level**	*Neisseria subflava* biovar *flava*	*Neisseria flavescens* (2.013)
		*Neisseria subflava* biovar *flava*	*Neisseria flavescens* (2.047)
		*Neisseria subflava* biovar *perflava*	*Neisseria flavescens* (2.036)
		*Neisseria subflava* biovar *subflava*	*Neisseria flavescens* (2.129
		*Neisseria sicca*	*Neisseria mucosa* (2.055))
		*Aeromonas hydrophila*	*Aeromonas caviae* (2.356)
		*Burkholderia cepacia*	*Burkholderia multivorans* (2.377)
		*Pseudomonas pseudoalcaligenes*	*Pseudomonas oleovorans* (2.2)
		*Shewanella algae*	*Shewanella putrefaciens* (2.143)
		*Corynebacterium pseudodiphtheriticum*	*Corynebacterium propinquum*(2.303)
		*Staphylococcus carnosus*	*Staphylococcus condimenti* (2.0)
		*Streptococcus australis*	*Streptococcus parasanguinis* (2.028)
		*Streptococcus australis*	*Streptococcus parasanguinis* (2.011)
		*Streptococcus infantarius*	*Streptococcus lutetiensis* (2.021)
		*Streptococcus infantis*	*Streptococcus peroris* (2.0)
	**Misidentification to genus level**	*Campylobacter hyointestinalis*	*Pandoraea sputorum* (2.213)
		*Rahnella aquatilis*	*Ewingella americana* (1.872)
		*Actinomyces naeslundii*	*Clostridium halophilum* (1.828)
		*Bacillus* sp.	*Corynebacterium tuberculostearicum* (2.02)
		*Microbacterium lacticum*	*Arthrobacter castelli* (2.154)
		*Candida freyschussii*	*Rhodotorula bogoriensis* (2.2)
**VITEK MS (IVD)**	**Misidentification to species level**	*Neisseria cinerea*	*Neisseria subflava* (99.9)
		*Burkholderia cepacia*	*Burkholderia multivorans* (99.9)
		*Fusarium proliferatum*	*Fusarium oxysporum* (97.6)
	**Misidentification to genus level**	*Campylobacter hyointestinalis*	*Comamonas testosteroni* (99.7)
		*Psychrobacter* sp.	*Eubacterium limosum* (99.8)
		*Corynebacterium* sp.	*Sphingobium xenophagum*(99.8)
		*Microbacterium aurum*	*Paenibacillus durus* (86.0)
		*Staphylococcus hominis*	*Kocuria kristinae* (99.9)
		*Gordonia bronchialis*	*Clavibacter michiganensis* (99.8)
		*Mycobacterium avium*	*Paenibacillus durus* (99.9)

^a^Bruker Biotyper confidence value is up to 3.0, and VITEK MS is up to 100.

**Table 4:** Identification results when the microorganism is present only in the VITEK MS IVD database.

Reference identification	Number of isolates	VITEK MS (IVD)				
		Correct identification to the level of				
		Species	Genus	Complex/group	No ID	Mis ID
Gram-positive cocci	1				1	
Other Gram-positive rods	2	2				
Non- fermentative Gram-negative rods	2	2				
Other Gram-negative bacteria	1	1				
Mycobacteriaceae	1	1				
Filamentous fungi	3	1		1	1	
Yeast	2	2				
**Total number of strains (%)**	**12**	**9 (75)**	**0**	**1 (8.3)**	**2 (16.7)**	**0**

No ID = No identification obtained. Mis ID = Misidentification obtained

For filamentous fungi, only 17 out of 71 strains tested were present in both dataset, but twice as much accounted for in Bruker database with 43 strains compared with 20 strains for VITEK MS IVD database. Overall Biotyper allowed the identification of 50.7% of the strains tested for that group of microorganism, as opposed to only 15.5% for VITEK MS. When present in their respective database, identification with Biotyper system yielded higher percentage of identification (to species, genus or complex) with 76.7% vs 50% correct identification on VITEK MS platform. Only one misidentification on the VITEK MS system (to the species level, see Table 3) was obtained when the filamentous fungus was initially present in the manufacturer’s database, and there was no misidentification for the Biotyper.

For yeast, slightly more of the 41 strains tested were present in Bruker database (35 strains) compared to VITEK IVD (30 strains). Overall performance of both systems was similar with 70.7% of yeast correctly identified to species, genus or complex level. That performance increased to 93.3% of correct identification for VITEK MS and to 82.9% for Biotyper when only the strains present in the manufacturer’s database were evaluated.

### Identification for Microorganisms Not Present in the Databases

Of the 642 strains tested, 70 were not referenced in the Bruker database at the species level while 249 were not in the VITEK MS IVD database used. Of this number, 71 mycobacteria and actinomycetes strains could not be tested with VITEK MS since the bioMérieux protocol failed to inactivate the strains. Of the 70 strains not present in the Bruker database at the species level, the Biotyper correctly identified 10 (14.3%) to the genus level and 2 (2.9%) to the complex/group level. For 52 (74.2%) strains, we obtained no identification and for 6 (8.6%) strains, an incorrect identification given (Table 5). Among those misidentifications, 2 were incorrect identification to the species level and 4 to the genus level. Of the 178 strains not present in the VITEK MS IVD database at the species level (excluding the 71 untested mycobacteria and actinomycetes), the VITEK MS correctly identified 12 (6.8%) of the strains each to the genus and to the complex/group level. For 97 (54.5%) strains, no identification was given and for 69 (38.7%) strains, an incorrect identification was obtained (Table 6). Among these incorrect identifications, 29 (16.2%) were incorrect identification to species level and 40 (22.5%) to genus level.

**Table 5:** Identification results when the microorganism is not present in the Bruker Biotyper database.

Reference identification	Number of isolates	Bruker Biotyper					Proposed IDs (confidence value)
		Misidentification to the species	Correct ID to the level of		No ID	Mis ID	
			Genus	Complex/group			
Gram-positive cocci							
*Arthrobacter soli*	1				1		
*Desemzia* sp.	1				1		
*Dolosigranulum pigrum*	1				1		
*Ignavigranum ruoffiae*	1				1		
*Micrococcus antarcticus*	1				1		
*Nosocomiicoccus ampullae*	1				1		
*Vagococcus carniphilus*	1				1		
**Total**	**7**	**0**	**0**	**0**	**7**	**0**	
Other Gram-positive rods							
*Corynebacterium massiliense*	1				1		
*Corynebacterium pyruviciproducens*	1				1		
*Microbacterium paraoxydans*	2		2				*Microbacterium sp*. (2.194)
							*Microbacterium sp*. (2.167)
*Terribacillus* sp.	1				1		
**Total**	**5**	**0**	**2**	**0**	**3**	**0**	
Enterobacteriaceae							
*Shigella boydii*	1					1	*Escherichia coli* (2.32)
*Shigella dysenteriae*	1					1	*Escherichia coli* (2.235)
*Shigella flexneri*	1					1	*Escherichia coli* (2.293)
*Shigella sonnei*	1					1	*Escherichia coli* (2.384)
**Total**	**4**	**0**	**0**	**0**	**0**	**4**	
Non-fermentative Gram-negative rods							
*Vibrio cholerae*	2		2				*Vibrio albensis* (1.897)
							*Vibrio albensis* (1.837)
**Total**	**2**	**0**	**2**	**0**	**0**	**0**	
Other Gram-negative bacteria							
*Haemophilus haemolyticus*	1				1		
*Lautropia mirabilis*	1				1		
**Total**	**2**	**0**	**0**	**0**	**2**	**0**	
Mycobacteriaceae							
*Mycobacterium elephantis*	1				1		
*Mycobacterium europaeum*	1				1		
*Mycobacterium goodii*	2	2					*Mycobacterium wolinskyi* (2.083)
							*Mycobacterium wolinskyi* (2.052)
*Mycobacterium kubicae*	1				1		
*Mycobacterium nebraskense*	1				1		
*Mycobacterium paraffinicum*	1				1		
*Mycobacterium parascrofulaceum*	3		2		1		*Mycobacterium avium* (1.903)
							*Mycobacterium scrofulaceum* (1.803)
*Mycobacterium porcinum*	1			1			
*Mycobacterium saskatchewanense*	1				1		
*Mycobacterium scrofulaceum*	1				1		
**Total**	**13**	**2**	**2**	**1**	**8**	**0**	
Actinomycetes							
*Actinomadura* sp.	1				1		
*Nocardia brasiliensis*	1		1				*Nocardia* sp. (1.755)
*Tsukamurella tyrosinosolvense*	1		1				*Tsukamurella paurometabola* (1,812)
**Total**	**3**	**0**	**2**	**0**	**1**	**0**	
Filamentous fungi							
*Acremonium strictum*	1				1		
*Aspergillus calidoustus*	1			1			
*Aspergillus insuetus*	1				1		
*Aspergillus melleus*	1		1				*Aspergillus ochraceus* (1.826)
*Aspergillus sclerotiorum*	1				1		
*Aspergillus sydowii*	1		1				*Aspergillus versicolor* (1.968)
*Cladosporium sphaeospermum*	1				1		
*Curvularia* sp.	1				1		
*Epicoccum* sp.	1				1		
*Geomyces pannorum*	1				1		
*Microsporum audouinii*	1				1		
*Neosartorya* sp.	1				1		
*Penicillium purpurogenum*	1				1		
*Phialemonium obvatum*	1				1		
*Phialophora verrucosa*	1				1		
*Pichia fabianii*	1				1		
*Pichia rhodanensis*	1				1		
*Pithomyces* sp.	1				1		
*Purpureocillium lavendulum*	1				1		
*Pyrenochaeta romeroi*	1				1		
*Scopulariopsis brumptii*	1				1		
*Syncephalastrum* sp.	1				1		
*Trichoderma* sp.	1				1		
*Trichophyton mentagrophytes*	1				1		
*Trichophyton soudanense*	1				1		
*Trichophyton terrestre*	1				1		
*Trichophyton verrucosum*	1				1		
*Trichophyton violaceum*	1				1		
**Total**	**28**	**0**	**2**	**1**	**25**	**0**	
Yeast							
*Candida bracarensis*	1				1		
*Candida tartarivorans*	1				1		
*Candida auris*	1				1		
*Cryptococcus terreus*	1				1		
*Cryptococcus albidus*	1				1		
*Lachancea fermentati*	1				1		
**Total**	**6**	**0**	**0**	**0**	**6**	**0**	
**Total number of strains (%)**	**70**	**2 (2.9)**	**10 (14.3)**	**2 (2.9)**	**52 (74.2)**	**4 (5.7)**	

No ID = No identification obtained. Mis ID = Misidentification obtained

**Table 6:** Identification results when the microorganism is not present in the VITEK MS IVD database.

Reference identification	Number of isolates	VITEK MS (IVD)					Proposed IDs (confidence value [%])
		Misidentification to the species	Correct ID to the level of		No ID	Mis ID	
			Genus	Complex/group			
Gram-positive cocci							
*Aerococcus sanguinicola*	1					1	*Streptococcus pluranimalium* (98.4)
*Desemzia* sp.	1					1	*Corynebacterium jeikeium* (95.7)
*Dolosigranulum pigrum*	1					1	*Paenibacillus peoriae* (99,9)
*Enterococcus malodoratus*	1	1					*Enterococcus avium* (99.9)
*Enterococcus phoeniculicola*	1				1		
*Facklamia languida*	1				1		
*Ignavigranum ruoffiae*	1					1	*Shewanella algae* (99.9)
*Kocuria marina*	1					1	*Serratia plymuthica* (91.4)
*Kocuria rhizophila*	1					1	*Kytococcus sedentarius* (99.9)
*Micrococcus antarcticus*	1	1					*Micrococcus luteus/lylae* (99.9)
*Nosocomiicoccus ampullae*	1					1	*Cronobacter malonaticus* (87.4)
*Rothia aeria*	3	3					*Rothia dentocariosa* (99.9)
							*Rothia dentocariosa* (99.9)
							*Rothia dentocariosa* (99.9)
*Rothia amarae*	1				1		
*Staphylococcus condimenti*	1	1					*Staphylococus carnosus* (99.6)
*Staphylococcus fleurettii*	1				1		
*Staphylococcus pettenkoferi*	1	1					*Staphylococcus auricularis* (92.3)
*Streptococcus australis*	2			2			
*Streptococcus infantis*	3	1		2			*Streptococcus pneumoniae* (99.9)
*Streptococcus pseudoporcinus*	5	5					*Streptococcus porcinus* (99.9)
							*Streptococcus porcinus* (99.9)
							*Streptococcus porcinus* (99.9)
							*Streptococcus porcinus* (99.9)
							*Streptococcus porcinus* (98.5)
*Vagococcus carniphilus*	1	1					*Vagococcus fluvialis* (99.9)
*Vagococcus lutrae*	1				1		
**Total**	**30**	**14**	**0**	**4**	**5**	**7**	
Anaerobes							
*Flavonifractor plautii*	1				1		
**Total**	**1**	**0**	**0**	**0**	**1**	**0**	
Other Gram-positive rods							
*Actinobaculum schaalii*	2					2	*Actinomyces meyeri* (99.7)
							*Yersinia enterocolitica* (99.8)
*Actinobaculum urinale*	1					1	*Streptococcus pseudopneumoniae* (98.9)
*Actinomyces canis*	1					1	*Micrococcus luteus/lylae* (97.6)
*Actinomyces graevenitzii*	2				2		
*Actinomyces naeslundii*	2	2					*Actinomyces viscosus* (99.9)
							*Actinomyces viscosus* (99.9)
*Actinomyces urogenitalis*	1				1		
*Atopobium rimae*	1					1	*Clostridium butyricum* (99.9)
*Bacillus flexus*	1	1					*Bacillus megaterium* (99.7)
*Bacillus halodurans*	1				1		
*Bacillus idriensis*	1				1		
*Bacillus nealsonii*	1				1		
*Bacillus siralis*	1				1		
*Corynebacterium accolens*	1				1		
*Corynebacterium afermentans*	1				1		
*Corynebacterium durum*	1				1		
*Corynebacterium massiliense*	1	1					*Corynebacterium tuberculostearicum* (99.9)
*Corynebacterium minutissimum*	3	2			1		*Corynebacterium aurimucosum* (99.9)
							*Corynebacterium aurimucosum* (99.9)
*Corynebacterium pyruviciproducens*	1				1		
*Corynebacterium riegelii*	1					1	*Burkholderia gladioli* (79.2)
*Curtobacterium* sp.	1					1	*Actinomyces meyeri* (99.9)
*Lactobacillus kalixensis*	1				1		
*Lactobacillus reuteri*	1				1		
*Paenibacillus taiwanensis*	1		1				*Paenibacillus* sp. (99.9)
*Propionimicrobium lymphophilum*	1					1	*Staphylococcus carnosus ssp carnosus* (99.9)
*Microbacterium lacticum*	2				1	1	*Paenibacillus durus* (99.9)
*Microbacterium oleivorans*	1				1		
*Sporosarcina* sp.	1				1		
*Terribacillus* sp.	1				1		
*Turicella otitidis*	2				2		
**Total**	**36**	**6**	**1**	**0**	**20**	**9**	
Enterobacteriaceae							
*Shigella boydii*	1					1	*Escherichia coli* (99.9)
*Shigella dysenteriae*	1					1	*Escherichia coli* (99.9)
*Shigella flexneri*	1					1	*Escherichia coli* (99.9)
*Shigella sonnei*	1					1	*Escherichia coli* (99.9)
**Total**	**4**	**0**	**0**	**0**	**0**	**4**	
Non-fermentative Gram-negative rods							
*Acidovorax temperans*	1				1		
*Burkholderia thailandensis*	1		1				*Burkholderia cepacia* (50.7)
*Burkholderia tropica*	1					1	*Yersinia ruckeri* (99.9)
*Capnocytophaga canimorsus*	1				1		
*Cupriavidus respiraculi*	1	1					*Cupriavidus pauculus* (96.8)
*Cupriavidus metallidurans*	1	1					*Cupriavidus pauculus* (99.9)
*Lautropia mirabilis*	1					1	*Haemophilus parainfluenzae* (99.7)
*Leptotrichia trevisanii*	1	1					*Leptotrichia buccalis* (99.9)
*Massilia timonae*	1				1		
*Pandoraea apista*	1				1		
*Pandoraea sputorum*	1				1		
*Pseudomonas pseudoalcaligenes*	1	1					*Pseudomonas oleovorans* (99.9)
*Roseomonas mucosa*	2				1	1	*Staphylococcus intermedius* (99.6)
**Total**	**14**	**4**	**1**	**0**	**6**	**3**	
Other Gram-negative bacteria							
*Arcobacter butzleri*	2				2		
*Bordetella holmesii*	1				1		
*Helicobacter cinaedi*	1					1	*Vibrio mimicus* (99.9)
*Legionella anisa*	3				3		
*Legionella bozemanae*	2		1		1		*Legionella pneumophila* (65.7)
*Legionella dumoffii*	1				1		
*Legionella jordanis*	1				1		
*Legionella longbeachae*	2				2		
*Legionella micdadei*	2				2		
*Legionella rubrilucens*	1				1		
*Moraxella atlantae*	1					1	*Eikenella corrodens* (78.4)
*Neisseria sicca*	1	1					*Neisseria mucosa* (99.9)
*Neisseria weaveri*	1	1					*Neisseria zoodegmatis* (99.9)
*Pasteurella bettyae*	1				1		
*Pasteurella dagmatis*	1					1	
**Total**	**21**	**2**	**1**	**0**	**15**	**3**	
Mycobacteriaceae							
*Mycobacterium conceptionense*	1				1		
*Mycobacterium florentinum*	1		1				*Mycobacterium tuberculosis* (50.0),
							*Mycobacterium bovis* (50.0)
*Mycobacterium gordonae*	1		1				*Mycobacterium tuberculosis* (50.0),
							*Mycobacterium bovis* (50.0)
*Mycobacterium interjectum*	1		1				*Mycobacterium tuberculosis* (50.0),
							*Mycobacterium bovis* (50.0)
*Mycobacterium kumamotonense*	1				1		
*Mycobacterium marinum*	1	1					*Mycobacterium kansasii* (86.5)
*Mycobacterium peregrinum*	1	1					*Mycobacterium fortuitum* (99.9)
*Mycobacterium porcinum*	1				1		
*Mycobacterium saskatchewanense*	1					1	*Staphylococcus vitulinus* (95.8)
**Total**	**9**	**2**	**3**	**0**	**3**	**1**	
Actinomycetes							
*Nocardia brasiliensis*	1				1		
*Tsukamurella tyrosinosolvense*	1				1		
**Total**	**2**	**0**	**0**	**0**	**2**	**0**	
Filamentous fungi							
*Acremonium strictum*	1				1		
*Arthrographis kalrae*	1				1		
*Aspergillus glaucus*	1				1		
*Aspergillus insuetus*	1			1			
*Aspergillus melleus*	1				1		
*Aspergillus sclerotiorum*	1					1	*Candida kefyr* (84.8)
*Aspergillus terreus*	1				1		
*Aureobasidium pullulans*	1				1		
*Chaetomium globosum*	1				1		
*Chrysosporium* sp.	2					2	*Candida famata* (99.9)
							*Candida norvegensis* (99.2)
*Cladosporium sphaeospermum*	1				1		
*Curvularia* sp.	1				1		
*Epicoccum* sp.	1					1	*Paecilomyces lilacinus* (99.8)
*Epidermophyton floccosum*	1				1		
*Exophiala dermatitidis*	1				1		
*Geomyces pannorum*	1					1	*Candida krusei* (97.1)
*Lichtheimia corymbifera*	1				1		
*Microsporum audouinii*	1				1		
*Microsporum canis*	1				1		
*Microsporum gypseum*	1				1		
*Microsporum persicolor*	1					1	*Candida kefyr* (85.1)
*Mucor* sp.	1				1		
*Neosartorya* sp.	1				1		
*Phialemonium obvatum*	1				1		
*Phialophora verrucosa*	1				1		
*Phoma* sp.	1					1	*Candida dubliniensis* (84.5)
*Pichia fabianii*	1				1		
*Pichia rhodanensis*	1					1	*Candida freyschussii* (99.9)
*Pithomyces* sp.	1				1		
*Pseudallescheria boydii*	1				1		
*Purpureocillium lavendulum*	1				1		
*Pyrenochaeta romeroi*	1				1		
*Rhizomucor pusillus*	1				1		
*Rhizopus stolonifer*	1					1	*Malassezia pachydermatis* (99.8)
*Scedosporium apiospermum*	2				2		
*Scedosporium prolificans*	1					1	*Williopsis saturnus* (96.2)
*Scopulariopsis brevicaulis*	1				1		
*Scopulariopsis brumptii*	1				1		
*Syncephalastrum* sp.	1				1		
*Trichoderma* sp.	1				1		
*Trichophyton interdigitale*	1					1	*Candida norvegica* (98.2)
*Trichophyton mentagrophytes*	1				1		
*Trichophyton rubrum*	2				2		
*Trichophyton soudanense*	1				1		
*Trichophyton terrestre*	1				1		
*Trichophyton tonsurans*	1				1		
*Trichophyton verrucosum*	1					1	*Candida tropicalis* (92.8)
*Trichophyton violaceum*	1				1		
**Total**	**51**	**0**	**0**	**1**	**38**	**12**	
Yeast							
*Candida bracarensis*	1					1	*Zygosaccharomyces bailii* (99.7)
*Candida nivariensis*	1				1		
*Candida orthopsilosis*	1			1			
*Candida pararugosa*	1				1		
*Candida tartarivorans*	1				1		
*Candida auris*	1				1		
*Candida palmioleophila*	1				1		
*Cryptococcus gattii*	1				1		
*Pseudozyma* sp.	1				1		
*Trichosporon cutaneum*	1	1					*Trichosporon mucoides* (99.8)
**Total**	**10**	**1**	**0**	**1**	**7**	**1**	
**Total number of strains (%)**	**178**	**29 (16.2)**	**6 (3.4)**	**6 (3.4)**	**97 (54.5)**	**40 (22.5)**	

No ID = No identification obtained. Mis ID = Misidentification obtained

For yeasts, a few of the strains tested were absent of the VITEK MS IVD database and Bruker database with 6 and 11 strains, respectively. The difference was even more pronounced for filamentous fungi with 51 strains not present in VITEK MS IVD compared to 28 for Bruker database. When filamentous fungi was absent from database, 12 misidentifications, mainly to genus, were recorded on the VITEK MS compared to none with Biotyper. Misidentification for yeast species occurred only twice with VITEK MS vs none with Biotyper.

### Impact of Protocols on Identification Performance

More than one extraction protocol can be used for bacterial identification. Among the bacteria analysed with each MALDI-TOF MS platform, a correct identification was obtained with the Bruker Biotyper for 199 out of the 364 bacteria tested (54.7%) with the direct colony protocol compared to 263 out of the 304 bacteria tested (86.5%) with the VITEK MS (*p<*0.0001). With the Bruker Biotyper, 98 identifications (26.9%) were obtained with the “on slide formic acid” extraction protocol. Then, 67 identifications (18.4%) were obtained with the “in tube formic acid” extraction protocol as the on slide acid formic protocol failed to provide suitable results (Table 7). We found that bacteria that needed formic acid extraction were Gram-positive isolates with mucoid or dry textured colonies.

**Table 7:** Correct identifications obtained for both MALDI-TOF MS technology according to the protocol used for bacteria isolates.

	Bruker Biotyper				VITEK MS (IVD)				
	Correct identification to the level of			Total (%)	Correct identification to the level of			Total (%)	*p* value
	Species	Genus	Complex/group		Species	Genus	Complex/group		
Direct colony	179	3	17	199 (54.7)	241	8	14	263 (86.5)	*p*<0.0001
On slide formic acid extraction	91	6	1	98 (26.9)	22	14	5	41 (13.5)	-
In tube formic acid extraction	28	38	1	67 (18.4)	NA	NA	NA	NA	-
**Total**	298	47	19	364	263	22	19	304	-

## Discussion

Only a few studies (two in Europe and one in USA) have published side by side evaluations comparing the performance of both systems on an extensive collection of various microorganisms [17–19]. These studies showed more than 90% overall species identification. Three groups also compared both systems specifically for the identification of yeasts [20–22]. Our study is the first performed in Canada, and also the first to include all varieties of microorganisms such as bacteria, mycobacteria, yeast and filamentous fungi. We obtained comparable results in our study, taking into account the correct identification to species, genus and complex/group combined. The findings of our study demonstrate that Bruker Biotyper and VITEK MS perform comparably for identification when the microorganism is present in the database. Analysed separately, the rate of correct identification to species, genus and complex/group for each microorganism’s category is similar for both systems, except for mycobacteria, actinomycetes and filamentous fungi. Bruker Biotyper was found to be superior for the identification of filamentous fungi with the current manufacturer’s database, with more strains accounted for in Bruker database, and fewer misidentifications obtained. However, VITEK MS correctly identified more mycobacteria and aerobic actinomycetes to species level compared to genus level identification for Bruker Biotyper. Some authors previously suggested that the score for the correct identification to species level of 2 for Biotyper could be lowered to 1.9 [23] or to 1.7 [24]. In our study, if the cut off used for species identification was 1.7, the ratio of correct mycobacteria and actinomycetes identifications to species obtained with the Bruker Biotyper system would have been comparable to VITEK MS. However, a large number of mycobacteria and actinomycetes strains have not been tested with VITEK MS since the inactivation protocol provided by bioMérieux at the time of the study was not functional and the strains were still viable after processing. This could have influenced the accurate comparison of both systems since a large number of strains tested only with the Bruker Biotyper system gave no identification. bioMérieux has since released (in August 2013) a new protocol for the inactivation of mycobacteria and actinomycetes. Other groups have suggested that the score for the correct identification to species level of 2 could be also lowered for other microorganisms such as certain species of *Staphylococcus*, *Streptococcus*, *Candida*, and dermatophytes [25–27]. Our results do not support these findings since species identification of these three genera were excellent with the actual cut off value, excepted for filamentous fungi.

As the LSPQ is the microbiology reference laboratory for the identification of microorganisms received from clinical laboratories in the province of Quebec, we possess a large collection of rare and unusual microorganisms. According to our knowledge, this study is the first to test the accuracy of identification of microorganisms not present in MALDI-TOF MS databases. For these microorganisms, the expected answer would have been no identification, or if a representative genus of the unknown strain was present, a correct identification to genus or complex/group. For this part of the study, VITEK MS gave significantly more misidentification than the Bruker Biotyper system. We recognize however that for more strains tested the name of the genus and / or species were not present in the VITEK MS IVD database compared to the Bruker database, since the RUO database was the only one available at the time of the study. This reflects the fact that the Bruker database contained more genus and species than the VITEK MS IVD database at the time of the evaluation. However, another database is available with the VITEK MS system (Saramis) which is an open database contained more species that the IVD database and which was not evaluated in this study. Consequently, the size and quality of the database are essential to accuracy and instrument performance. For Bruker Biotyper system, the four misidentifications to the genus level were four strains of *Shigella* identified as *E*. *coli*. The limitation of identification for some microorganisms are well known and transparently mentioned by the manufacturer. The only two other misidentifications by the Bruker Biotyper system were two strains of *Mycobacterium goodii* identified as *Mycobacterium wolinskyi*. This two species share similar biochemical and growth characteristics and are both closely related to *M*. *smegmatis* [28]. VITEK MS gave a worrying number of misidentifications when the microorganism was not in the database, particularly for Gram-positive cocci and rods, non-fermentative Gram-negative rods and filamentous fungi. Nearly half of the misidentifications were to the species level, the remaining misidentifications being most problematic, except for the *Shigella* strains which are a known limitation of the system. In addition, some misidentifications to the genus are from the opposite Gram stain, reinforcing the fact that Gram staining and morphology examination should still be maintained in an identification algorithm comprising MALDI-TOF MS. Six out of nine non tuberculous mycobacteria (NTM) were wrongly identified at the species or the genus level. The fact that 3 NTM were considered as members of the *M*. *tuberculosis* complex, even with low confidence, may poses a problem in patient management treatment. However, the protocol used at the time of the study could also have influenced these misidentifications. Misidentification of filamentous fungi with VITEK MS appears particularly problematic, with 12 misidentifications which were given an incorrect yeast genus.

Authors in other MALDI-TOF MS evaluation studies have previously reported the use of a formic acid-based protein extraction using a bacterial lysis step or the use of formic acid directly on the bacterial smear before matrix application to be needed, mainly for Gram-positive bacteria. This was mainly reported in studies using the Bruker Biotyper system [29–33]. We observed that formic acid extraction methods, both on slide or in tube, were used significantly more to obtain accurate identification with the Bruker Biotyper system than the VITEK MS system. This is an important issue since repeating the identification with the formic acid extraction method, particularly with the in tube protocol, could delay the time of results. As shown by Dubois et al, the use of the VITEK MS system generated a low frequency of unusable spectra without formic acid use that is compatible and convenient for routine practice [34]. Deak *et al*. reported that Bruker Biotyper system was extremely sensitive to the amount of organism applied to the target plate, and over-application often resulted in low confidence scores or no identification, particularly for yeasts [35]. They also reported that in contrast, VITEK MS produces accurate results for yeast with high percent confidence levels for species identification done through formic acid extraction performed directly on the target plate. According to our experience, the use of a prior formic acid extraction step, otherwise than recommended by the manufacturer protocols, should be used with Gram-positive isolates with mucoid or dry textured colonies for MALDI TOF MS identification.

## Conclusions

In conclusion, our study demonstrates that both systems gave a high level (above 85%) of correct identification for a wide range of microorganisms. However, VITEK MS gave more misidentification when the microorganism analysed was not present in the database, compared to Bruker Biotyper. This should be taken into account for the utilisation of this technology alone for microorganism identification in a public health laboratory, where isolates received are often difficult to identify and/or unusual microorganisms. Both systems allow the user to add his own spectra in order to build his own database (for VITEK MS it is in a separate database called SARAMIS). In a public health reference laboratory, validation of a species that increasing database content should allow to raise the identification power of MALDI-TOF MS.

## Supporting Information

S1 Table Complete list of microorganism identification results when the microorganism is present in both databases. (DOCX)

S2 Table Complete list of microorganism identification results when the microorganism is present only in the Bruker Biotyper database. (DOCX)

S3 Table Complete list of microorganism identification results when the microorganism is present only in the VITEK MS IVD database. (DOCX)
