# Supplementary material for: A Side by Side Comparison of Bruker Biotyper and VITEK MS: Utility of MALDI-TOF MS Technology for Microorganism Identification in a Public Health Reference Laboratory
Source: PLoS One. 2015 Dec 10;10(12):e0144878. doi: 10.1371/journal.pone.0144878 (PMC4689555; doi:10.1371/journal.pone.0144878)
Supplement: S1 Table — (DOCX) [file pone.0144878.s001.docx]

**S1Table. Complete list of microorganism identification results when the microorganism is present in both databases.**

| **Reference identification** | **Number of isolates** | **Bruker Biotyper** | | | | | **VITEK MS (IVD)** | | | | |
| --- | --- | --- | --- | --- | --- | --- | --- | --- | --- | --- | --- |
|  |  | **Correct identification to the level of** | | |  |  | **Correct identification to the level of** | | |  |  |
|  |  | **Species** | **Genus** | **Complex/group** | **No ID** | **Mis ID** | **Species** | **Genus** | **Complex/group** | **No ID** | **Mis ID** |
| Gram-positive cocci |  |  |  |  |  |  |  |  |  |  |  |
| *Abiotrophia defectiva* | 3 | 3 |  |  |  |  | 3 |  |  |  |  |
| *Aerococcus urinae* | 3 | 3 |  |  |  |  | 3 |  |  |  |  |
| *Aerococcus viridans* | 2 | 1 | 1 |  |  |  | 2 |  |  |  |  |
| *Dermacoccus nishinomiyaensis* | 1 | 1 |  |  |  |  | 1 |  |  |  |  |
| *Dermacoccus* sp. | 2 |  |  |  | 2 |  |  | 1 |  | 1 |  |
| *Enterococcus avium* | 2 | 2 |  |  |  |  | 2 |  |  |  |  |
| *Enterococcus casseliflavus* | 2 | 2 |  |  |  |  | 2 |  |  |  |  |
| *Enterococcus durans* | 2 | 2 |  |  |  |  | 2 |  |  |  |  |
| *Enterococcus faecalis* | 2 | 2 |  |  |  |  | 2 |  |  |  |  |
| *Enterococcus faecium* | 2 | 2 |  |  |  |  | 2 |  |  |  |  |
| *Enterococcus gallinarum* | 2 | 2 |  |  |  |  | 2 |  |  |  |  |
| *Enterococcus hirae* | 2 | 2 |  |  |  |  | 2 |  |  |  |  |
| *Enterococcus raffinosus* | 2 | 2 |  |  |  |  | 2 |  |  |  |  |
| *Facklamia hominis* | 1 | 1 |  |  |  |  | 1 |  |  |  |  |
| *Gemella bergeri* | 1 | 1 |  |  |  |  | 1 |  |  |  |  |
| *Gemella haemolysans* | 1 |  | 1 |  |  |  | 1 |  |  |  |  |
| *Gemella morbillorum* | 1 | 1 |  |  |  |  | 1 |  |  |  |  |
| *Gemella sanguinis* | 1 |  | 1 |  |  |  | 1 |  |  |  |  |
| *Globicatella sulfidifaciens* | 1 | 1 |  |  |  |  |  | 1 |  |  |  |
| *Granulicatella adiacens* | 2 | 2 |  |  |  |  | 2 |  |  |  |  |
| *Granulicatella elegans* | 1 |  | 1 |  |  |  | 1 |  |  |  |  |
| *Helcococcus kunzii* | 2 | 2 |  |  |  |  | 2 |  |  |  |  |
| *Kocuria kristinae* | 1 | 1 |  |  |  |  | 1 |  |  |  |  |
| *Kocuria rosea* | 1 | 1 |  |  |  |  | 1 |  |  |  |  |
| *Lactococcus garvieae* | 2 | 2 |  |  |  |  | 2 |  |  |  |  |
| *Lactococcus lactis* | 2 | 2 |  |  |  |  | 2 |  |  |  |  |
| *Leuconostoc citreum* | 1 | 1 |  |  |  |  | 1 |  |  |  |  |
| *Micrococcus luteus* | 2 | 2 |  |  |  |  | 2 |  |  |  |  |
| *Rothia dentocariosa* | 1 | 1 |  |  |  |  | 1 |  |  |  |  |
| *Rothia mucilaginosa* | 1 | 1 |  |  |  |  | 1 |  |  |  |  |
| *Staphylococcus aureus* | 5 | 5 |  |  |  |  | 5 |  |  |  |  |
| *Staphylococcus capitis* | 1 | 1 |  |  |  |  | 1 |  |  |  |  |
| *Staphylococcus caprae* | 1 | 1 |  |  |  |  | 1 |  |  |  |  |
| *Staphylococcus carnosus* | 1 |  |  |  |  | 1 | 1 |  |  |  |  |
| *Staphylococcus cohnii* | 1 | 1 |  |  |  |  | 1 |  |  |  |  |
| *Staphylococcus hominis* | 1 |  | 1 |  |  |  |  |  |  |  | 1 |
| *Staphylococcus lugdunensis* | 1 | 1 |  |  |  |  | 1 |  |  |  |  |
| *Staphylococcus pseudintermedius* | 1 | 1 |  |  |  |  | 1 |  |  |  |  |
| *Staphylococcus saccharolyticus* | 1 |  |  |  | 1 |  | 1 |  |  |  |  |
| *Staphylococcus saprophyticus* | 1 | 1 |  |  |  |  | 1 |  |  |  |  |
| *Staphylococcus simulans* | 1 | 1 |  |  |  |  | 1 |  |  |  |  |
| *Staphylococcus warneri* | 1 | 1 |  |  |  |  | 1 |  |  |  |  |
| *Streptococcus anginosus* | 1 | 1 |  |  |  |  | 1 |  |  |  |  |
| *Streptococcus constellatus* | 1 | 1 |  |  |  |  | 1 |  |  |  |  |
| *Streptococcus cristatus* | 1 | 1 |  |  |  |  | 1 |  |  |  |  |
| *Streptococcus dysgalactiae* | 2 | 2 |  |  |  |  |  | 2 |  |  |  |
| *Streptococcus gallolyticus* | 3 | 3 |  |  |  |  | 3 |  |  |  |  |
| *Streptococcus gordonii* | 2 | 2 |  |  |  |  | 2 |  |  |  |  |
| *Streptococcus infantarius* | 1 |  |  |  |  | 1 | 1 |  |  |  |  |
| *Streptococcus intermedius* | 2 |  | 2 |  |  |  | 2 |  |  |  |  |
| *Streptococcus lutetiensis* | 3 | 3 |  |  |  |  | 2 | 1 |  |  |  |
| *Streptococcus* MOP group | 1 |  |  | 1 |  |  |  |  | 1 |  |  |
| *Streptococcus mutans* | 1 | 1 |  |  |  |  | 1 |  |  |  |  |
| *Streptococcus oralis* | 1 |  |  | 1 |  |  |  |  | 1 |  |  |
| *Streptococcus parasanguinis* | 2 | 2 |  |  |  |  | 2 |  |  |  |  |
| *Streptococcus pasteurianus* | 2 | 2 |  |  |  |  | 2 |  |  |  |  |
| *Streptococcus pneumoniae* | 11 |  |  | 11 |  |  | 11 |  |  |  |  |
| *Streptococcus salivarius* | 2 | 2 |  |  |  |  | 1 | 1 |  |  |  |
| *Streptococcus sanguinis* | 1 | 1 |  |  |  |  | 1 |  |  |  |  |
| *Streptococcus vestibularis* | 1 | 1 |  |  |  |  |  | 1 |  |  |  |
| *Vagococcus fluvialis* | 1 | 1 |  |  |  |  | 1 |  |  |  |  |
| **Total** | **104** | **79** | **7** | **13** | **3** | **2** | **93** | **7** | **2** | **1** | **1** |
|  |  |  |  |  |  |  |  |  |  |  |  |
| Anaerobes |  |  |  |  |  |  |  |  |  |  |  |
| *Clostridium difficile* | 4 | 4 |  |  |  |  | 4 |  |  |  |  |
| *Clostridium tertium* | 1 | 1 |  |  |  |  | 1 |  |  |  |  |
| **Total** | **5** | **5** | **0** | **0** | **0** | **0** | **5** | **0** | **0** | **0** | **0** |
|  |  |  |  |  |  |  |  |  |  |  |  |
| Other Gram-positive rods |  |  |  |  |  |  |  |  |  |  |  |
| *Actinomyces meyeri* | 2 | 2 |  |  |  |  | 2 |  |  |  |  |
| *Actinomyces neuii* | 1 | 1 |  |  |  |  | 1 |  |  |  |  |
| *Actinomyces odontolyticus* | 3 | 3 |  |  |  |  | 2 | 1 |  |  |  |
| *Actinomyces radingae* | 2 | 2 |  |  |  |  | 2 |  |  |  |  |
| *Actinomyces turicensis* | 2 | 2 |  |  |  |  | 2 |  |  |  |  |
| *Aneurinibacillus aneurinilyticus* | 1 | 1 |  |  |  |  | 1 |  |  |  |  |
| *Arcanobacterium haemolyticum* | 1 | 1 |  |  |  |  | 1 |  |  |  |  |
| *Arthrobacter* sp. | 1 |  | 1 |  |  |  |  |  |  | 1 |  |
| *Bacillus cereus* group | 2 |  |  | 2 |  |  |  | 2 |  |  |  |
| *Bacillus licheniformis* | 2 | 1 | 1 |  |  |  | 1 |  |  | 1 |  |
| *Bacillus megaterium* | 1 | 1 |  |  |  |  | 1 |  |  |  |  |
| *Bacillus mycoides* | 1 | 1 |  |  |  |  |  |  | 1 |  |  |
| *Bacillus pumilus* | 1 | 1 |  |  |  |  | 1 |  |  |  |  |
| *Bacillus* sp. | 3 |  | 1 |  | 1 | 1 |  | 2 |  | 1 |  |
| *Bacillus subtilis* | 2 | 1 | 1 |  |  |  |  | 2 |  |  |  |
| *Bifidobacterium breve^b^* | 1 |  |  |  | 1 |  |  | 1 |  |  |  |
| *Bifidobacterium scardovii^b^* | 2 | 2 |  |  |  |  |  | 2 |  |  |  |
| *Brevibacillus laterosporus^c^* | 1 | 1 |  |  |  |  |  | 1 |  |  |  |
| *Brevibacillus reuszeri^c^* | 1 |  | 1 |  |  |  |  | 1 |  |  |  |
| *Brevibacterium casei* | 1 | 1 |  |  |  |  | 1 |  |  |  |  |
| *Cellulosimicrobium* sp. | 1 |  | 1 |  |  |  |  | 1 |  |  |  |
| *Corynebacterium amycolatum* | 1 | 1 |  |  |  |  |  | 1 |  |  |  |
| *Corynebacterium aurimucosum* | 2 | 2 |  |  |  |  | 1 | 1 |  |  |  |
| *Corynebacterium auris* | 1 |  | 1 |  |  |  | 1 |  |  |  |  |
| *Corynebacterium jeikeium* | 2 | 2 |  |  |  |  | 2 |  |  |  |  |
| *Corynebacterium mucifaciens* | 1 | 1 |  |  |  |  | 1 |  |  |  |  |
| *Corynebacterium pseudodiphtheriticum* | 2 | 1 |  |  |  | 1 | 2 |  |  |  |  |
| *Corynebacterium simulans* | 2 | 2 |  |  |  |  | 2 |  |  |  |  |
| *Corynebacterium* sp. | 2 |  | 1 |  | 1 |  |  |  |  | 1 | 1 |
| *Corynebacterium tuberculostearicum* | 1 | 1 |  |  |  |  | 1 |  |  |  |  |
| *Corynebacterium urealyticum* | 1 |  | 1 |  |  |  | 1 |  |  |  |  |
| *Dermabacter hominis* | 2 | 1 | 1 |  |  |  | 2 |  |  |  |  |
| *Dietzia cinnamea* | 1 |  |  |  | 1 |  | 1 |  |  |  |  |
| *Lactobacillus casei / paracasei* | 1 |  |  | 1 |  |  |  |  | 1 |  |  |
| *Lactobacillus crispatus* | 1 | 1 |  |  |  |  | 1 |  |  |  |  |
| *Lactobacillus delbruckii* | 1 | 1 |  |  |  |  | 1 |  |  |  |  |
| *Lactobacillus fermentum* | 2 | 2 |  |  |  |  | 2 |  |  |  |  |
| *Lactobacillus gasseri* | 1 | 1 |  |  |  |  |  |  | 1 |  |  |
| *Lactobacillus rhamnosus* | 1 | 1 |  |  |  |  | 1 |  |  |  |  |
| *Listeria monocytogenes* | 1 | 1 |  |  |  |  | 1 |  |  |  |  |
| *Lysinibacillus sphaericus* | 1 | 1 |  |  |  |  |  | 1 |  |  |  |
| *Microbacterium aurum* | 1 | 1 |  |  |  |  |  |  |  |  | 1 |
| *Microbacterium* sp. | 1 |  | 1 |  |  |  |  | 1 |  |  |  |
| *Paenibacillus* sp. | 1 |  | 1 |  |  |  |  | 1 |  |  |  |
| *Propionibacterium acnes* | 2 | 2 |  |  |  |  | 2 |  |  |  |  |
| *Propionibacterium avidum* | 2 | 2 |  |  |  |  | 1 | 1 |  |  |  |
| *Propionibacterium propionicum* | 1 |  |  |  | 1 |  | 1 |  |  |  |  |
| *Rothia dentocariosa* | 3 | 3 |  |  |  |  | 3 |  |  |  |  |
| *Trueperalla bernardiae* | 5 | 5 |  |  |  |  | 5 |  |  |  |  |
| **Total** | **75** | **53** | **12** | **3** | **5** | **2** | **47** | **19** | **3** | **4** | **2** |
|  |  |  |  |  |  |  |  |  |  |  |  |
| Enterobacteriaceae |  |  |  |  |  |  |  |  |  |  |  |
| *Citrobacter braakii* | 1 | 1 |  |  |  |  | 1 |  |  |  |  |
| *Citrobacter freundii* | 1 | 1 |  |  |  |  | 1 |  |  |  |  |
| *Citrobacter koseri* | 1 | 1 |  |  |  |  | 1 |  |  |  |  |
| *Enterobacter aerogenes* | 1 | 1 |  |  |  |  | 1 |  |  |  |  |
| *Pantoae agglomerans* | 1 |  |  |  | 1 |  | 1 |  |  |  |  |
| *Escherichia coli* | 1 | 1 |  |  |  |  | 1 |  |  |  |  |
| *Escherichia vulneris* | 1 | 1 |  |  |  |  | 1 |  |  |  |  |
| *Listeria monocytogenes* | 1 |  |  | 1 |  |  |  |  | 1 |  |  |
| *Hafnia alvei* | 1 | 1 |  |  |  |  | 1 |  |  |  |  |
| *Klebsiella oxytoca* | 2 | 2 |  |  |  |  | 2 |  |  |  |  |
| *Morganella morganii* | 1 | 1 |  |  |  |  | 1 |  |  |  |  |
| *Salmonella enterica* | 1 |  | 1 |  |  |  |  | 1 |  |  |  |
| *Shewanella algae* | 1 |  |  |  |  | 1 | 1 |  |  |  |  |
| *Yersinia enterocolitica* | 1 | 1 |  |  |  |  | 1 |  |  |  |  |
| *Yersinia intermedia* | 1 | 1 |  |  |  |  | 1 |  |  |  |  |
| *Yersinia pseudotuberculosis* | 1 | 1 |  |  |  |  | 1 |  |  |  |  |
| **Total** | **17** | **13** | **1** | **1** | **1** | **1** | **15** | **1** | **1** | **0** | **0** |
|  |  |  |  |  |  |  |  |  |  |  |  |
| Non-fermentative Gram-negative rods |  |  |  |  |  |  |  |  |  |  |  |
| *Achromobacter xylosoxidans* | 1 | 1 |  |  |  |  |  | 1 |  |  |  |
| *Acinetobacter baumannii* | 1 | 1 |  |  |  |  |  |  | 1 |  |  |
| *Acinetobacter calcoaceticus* | 1 | 1 |  |  |  |  |  |  | 1 |  |  |
| *Acinetobacter johnsonii* | 1 | 1 |  |  |  |  | 1 |  |  |  |  |
| *Acinetobacter junii* | 1 | 1 |  |  |  |  | 1 |  |  |  |  |
| *Acinetobacter radioresistens* | 1 | 1 |  |  |  |  | 1 |  |  |  |  |
| *Acinetobacter ursingii* | 1 | 1 |  |  |  |  | 1 |  |  |  |  |
| *Aeromonas hydrophila* | 1 |  |  |  |  | 1 |  |  | 1 |  |  |
| *Aeromonas veronii* | 1 | 1 |  |  |  |  |  | 1 |  |  |  |
| *Bergeyella zoohelcum* | 1 | 1 |  |  |  |  | 1 |  |  |  |  |
| *Agrobacterium radiobacter* | 1 | 1 |  |  |  |  | 1 |  |  |  |  |
| *Brevundimonas vesicularis* | 1 |  | 1 |  |  |  | 1 |  |  |  |  |
| *Burkholderia cepacia* | 1 |  |  |  |  | 1 |  |  |  |  | 1 |
| *Burkholderia gladioli* | 1 | 1 |  |  |  |  | 1 |  |  |  |  |
| *Burkholderia multivorans* | 1 | 1 |  |  |  |  | 1 |  |  |  |  |
| *Capnocytophaga sputigena* | 1 | 1 |  |  |  |  | 1 |  |  |  |  |
| *Cardiobacterium hominis* | 1 |  |  |  | 1 |  | 1 |  |  |  |  |
| *Comamonas testosteroni* | 1 | 1 |  |  |  |  | 1 |  |  |  |  |
| *Cupriavidus pauculus* | 1 | 1 |  |  |  |  | 1 |  |  |  |  |
| *Delftia acidovorans* | 1 | 1 |  |  |  |  | 1 |  |  |  |  |
| *Fusobacterium nucleatum* | 1 | 1 |  |  |  |  | 1 |  |  |  |  |
| *Gardnerella vaginalis* | 1 | 1 |  |  |  |  | 1 |  |  |  |  |
| *Inquilinus limosus* | 1 |  |  |  | 1 |  |  |  |  | 1 |  |
| *Leclercia adecarboxylata* | 1 | 1 |  |  |  |  | 1 |  |  |  |  |
| *Methylobacterium sp.* | 1 |  |  |  | 1 |  |  |  |  | 1 |  |
| *Myroides odoratus^a^* | 1 | 1 |  |  |  |  |  | 1 |  |  |  |
| *Ochrobactrum anthropi* | 1 | 1 |  |  |  |  | 1 |  |  |  |  |
| *Oligella urethralis* | 1 | 1 |  |  |  |  | 1 |  |  |  |  |
| *Pseudomonas aeruginosa* | 1 | 1 |  |  |  |  | 1 |  |  |  |  |
| *Pseudomonas fluorescens* | 1 |  |  | 1 |  |  | 1 |  |  |  |  |
| *Pseudomonas putida* | 1 |  |  | 1 |  |  | 1 |  |  |  |  |
| *Pseudomonas stutzeri* | 1 | 1 |  |  |  |  | 1 |  |  |  |  |
| *Psychrobacter* sp. | 1 |  |  |  | 1 |  |  |  |  |  | 1 |
| *Rahnella aquatilis* | 1 |  |  |  |  | 1 | 1 |  |  |  |  |
| *Ralstonia insidiosa* | 1 | 1 |  |  |  |  | 1 |  |  |  |  |
| *Ralstonia mannitolilytica* | 1 | 1 |  |  |  |  | 1 |  |  |  |  |
| *Sphingobacterium multivorum* | 1 | 1 |  |  |  |  | 1 |  |  |  |  |
| *Sphingomonas paucimobilis* | 1 | 1 |  |  |  |  | 1 |  |  |  |  |
| [*Stenotrophomonas maltophilia*](file:///C:\Documents%20and%20Settings\levsim01\Local%20Settings\Temporary%20Internet%20Files\Content.MSO\9836AC79.xlsx#RANGE!ID0EVCA) | 1 | 1 |  |  |  |  | 1 |  |  |  |  |
| *Vibrio alginolyticus* | 1 | 1 |  |  |  |  | 1 |  |  |  |  |
| *Vibrio fluvialis* | 1 | 1 |  |  |  |  | 1 |  |  |  |  |
| *Vibrio parahaemolyticus* | 1 | 1 |  |  |  |  | 1 |  |  |  |  |
| **Total** | **42** | **32** | **1** | **2** | **4** | **3** | **32** | **3** | **3** | **2** | **2** |
|  |  |  |  |  |  |  |  |  |  |  |  |
| Other Gram-negative bacteria |  |  |  |  |  |  |  |  |  |  |  |
| *Aggregatibacter actinomycetemcomitans* | 1 | 1 |  |  |  |  | 1 |  |  |  |  |
| *Aggregatibacter aphrophilus* | 1 | 1 |  |  |  |  | 1 |  |  |  |  |
| *Aggregatibacter segnis* | 1 | 1 |  |  |  |  | 1 |  |  |  |  |
| *Bordetella bronchiseptica* | 1 | 1 |  |  |  |  | 1 |  |  |  |  |
| *Bordetella parapertussis* | 2 | 2 |  |  |  |  | 2 |  |  |  |  |
| *Bordetella pertussis* | 4 | 4 |  |  |  |  | 4 |  |  |  |  |
| *Campylobacter coli* | 2 | 2 |  |  |  |  | 2 |  |  |  |  |
| *Campylobacter fetus* | 2 | 2 |  |  |  |  | 2 |  |  |  |  |
| *Campylobacter hyointestinalis* | 1 |  |  |  |  | 1 |  |  |  |  | 1 |
| *Campylobacter jejuni* | 1 | 1 |  |  |  |  | 1 |  |  |  |  |
| *Campylobacter lari* | 1 | 1 |  |  |  |  | 1 |  |  |  |  |
| *Campylobacter upsaliensis* | 2 | 2 |  |  |  |  | 2 |  |  |  |  |
| *Campylobacter ureolyticus* | 1 | 1 |  |  |  |  | 1 |  |  |  |  |
| *Eikenella corrodens* | 1 | 1 |  |  |  |  | 1 |  |  |  |  |
| *Haemophilus influenzae* | 5 | 5 |  |  |  |  | 5 |  |  |  |  |
| *Haemophilus parahaemolyticus* | 1 | 1 |  |  |  |  | 1 |  |  |  |  |
| *Haemophilus parainfluenzae* | 1 | 1 |  |  |  |  | 1 |  |  |  |  |
| *Kingella denitrificans* | 1 |  | 1 |  |  |  | 1 |  |  |  |  |
| *Kingella kingae* | 1 | 1 |  |  |  |  | 1 |  |  |  |  |
| *Legionella pneumophila* | 6 | 6 |  |  |  |  | 6 |  |  |  |  |
| *Moraxella catarrhalis* | 1 | 1 |  |  |  |  | 1 |  |  |  |  |
| *Moraxella lacunata* | 1 |  | 1 |  |  |  | 1 |  |  |  |  |
| *Moraxella nonliquefaciens* | 1 | 1 |  |  |  |  | 1 |  |  |  |  |
| *Moraxella osloensis* | 1 |  | 1 |  |  |  | 1 |  |  |  |  |
| *Neisseria cinerea* | 2 | 1 | 1 |  |  |  | 1 |  |  |  | 1 |
| *Neisseria elongata* | 1 |  | 1 |  |  |  | 1 |  |  |  |  |
| *Neisseria gonorrhoeae* | 5 | 5 |  |  |  |  | 5 |  |  |  |  |
| *Neisseria lactamica* | 2 | 2 |  |  |  |  | 2 |  |  |  |  |
| *Neisseria meningitidis* | 7 | 7 |  |  |  |  | 7 |  |  |  |  |
| *Neisseria mucosa* | 2 | 1 | 1 |  |  |  | 2 |  |  |  |  |
| *Neisseria subflava* biovar *flava* | 2 |  |  |  |  | 2 | 2 |  |  |  |  |
| *Neisseria subflava* biovar *perflava* | 2 | 1 |  |  |  | 1 | 2 |  |  |  |  |
| *Neisseria subflava* biovar *subflava* | 1 |  |  |  |  | 1 | 1 |  |  |  |  |
| *Pasteurella canis* | 1 | 1 |  |  |  |  | 1 |  |  |  |  |
| *Pasteurella multocida* | 1 | 1 |  |  |  |  | 1 |  |  |  |  |
| **Total** | **66** | **55** | **6** | **0** | **0** | **5** | **64** | **0** | **0** | **0** | **2** |
|  |  |  |  |  |  |  |  |  |  |  |  |
| Mycobacteriaceae |  |  |  |  |  |  |  |  |  |  |  |
| *Mycobacterium avium* | 4 |  | 4 |  |  |  | 3 |  |  |  | 1 |
| *Mycobacterium fortuitum* | 2 | 1 | 1 |  |  |  | 1 | 1 |  |  |  |
| *Mycobacterium intracellulare* | 4 | 2 | 2 |  |  |  | 4 |  |  |  |  |
| *Mycobacterium kansasii* | 8 | 2 | 6 |  |  |  | 8 |  |  |  |  |
| **Total** | **18** | **5** | **13** | **0** | **0** | **0** | **16** | **1** | **0** | **0** | **1** |
|  |  |  |  |  |  |  |  |  |  |  |  |
| Actinomycetes |  |  |  |  |  |  |  |  |  |  |  |
| *Dietzia cinnamea* | 2 |  |  |  | 2 |  | 2 |  |  |  |  |
| *Gordonia bronchialis* | 2 | 1 | 1 |  |  |  | 1 |  |  |  | 1 |
| *Gordonia* sp. | 1 |  |  |  | 1 |  |  |  |  | 1 |  |
| *Rhodococcus fascians* | 2 | 2 |  |  |  |  | 2 |  |  |  |  |
| *Rhodococcus* sp. | 1 |  | 1 |  |  |  |  | 1 |  |  |  |
| **Total** | **8** | **3** | **2** | **0** | **3** | **0** | **5** | **1** | **0** | **1** | **1** |
|  |  |  |  |  |  |  |  |  |  |  |  |
| Filamentous fungi |  |  |  |  |  |  |  |  |  |  |  |
| *Alternaria sp.* | 1 |  |  |  | 1 |  |  | 1 |  |  |  |
| *Arthrinium* sp. | 1 |  | 1 |  |  |  |  |  |  | 1 |  |
| *Aspergillus flavipes* | 1 | 1 |  |  |  |  |  |  |  | 1 |  |
| *Aspergillus flavus* | 1 | 1 |  |  |  |  | 1 |  |  |  |  |
| *Aspergillus fumigatus* | 3 | 3 |  |  |  |  | 2 |  |  | 1 |  |
| *Aspergillus nidulans* | 1 | 1 |  |  |  |  |  |  |  | 1 |  |
| *Aspergillus niger* | 1 | 1 |  |  |  |  | 1 |  |  |  |  |
| *Aspergillus ochraceus* | 1 |  | 1 |  |  |  |  |  |  | 1 |  |
| *Aspergillus versicolor* | 1 |  | 1 |  |  |  |  |  |  | 1 |  |
| *Fusarium proliferatum* | 1 | 1 |  |  |  |  |  |  |  |  | 1 |
| *Geotrichum capitatum* | 1 | 1 |  |  |  |  | 1 |  |  |  |  |
| *Paecilomyces variotii* | 1 | 1 |  |  |  |  | 1 |  |  |  |  |
| *Penicillium* sp. | 2 |  | 1 |  | 1 |  |  |  |  | 2 |  |
| *Purpureocillium lilacinus* | 1 | 1 |  |  |  |  | 1 |  |  |  |  |
| **Total** | **17** | **11** | **4** | **0** | **2** | **0** | **7** | **1** | **0** | **8** | **1** |
|  |  |  |  |  |  |  |  |  |  |  |  |
| Yeast |  |  |  |  |  |  |  |  |  |  |  |
| *Candida albicans* | 1 | 1 |  |  |  |  | 1 |  |  |  |  |
| *Candida dubliniensis* | 1 | 1 |  |  |  |  | 1 |  |  |  |  |
| *Candida freyschussii* | 1 |  |  |  |  | 1 | 1 |  |  |  |  |
| *Candida glabrata* | 2 | 2 |  |  |  |  | 2 |  |  |  |  |
| *Candida guilliermondii* | 1 | 1 |  |  |  |  | 1 |  |  |  |  |
| *Candida haemulonii* | 1 | 1 |  |  |  |  | 1 |  |  |  |  |
| *Candida krusei* | 1 | 1 |  |  |  |  | 1 |  |  |  |  |
| *Candida lusitaniae* | 2 | 2 |  |  |  |  | 2 |  |  |  |  |
| *Candida norvegensis* | 1 | 1 |  |  |  |  | 1 |  |  |  |  |
| *Candida parapsilosis* | 2 | 2 |  |  |  |  | 2 |  |  |  |  |
| *Candida tropicalis* | 2 | 2 |  |  |  |  | 2 |  |  |  |  |
| *Candida zeylanoides* | 1 | 1 |  |  |  |  | 1 |  |  |  |  |
| *Cryptococcus laurentii* | 1 |  |  |  | 1 |  | 1 |  |  |  |  |
| *Cryptococcus neoformans* | 3 | 3 |  |  |  |  | 3 |  |  |  |  |
| *Kodamaea ohmeri* | 1 | 1 |  |  |  |  | 1 |  |  |  |  |
| *Malassezia furfur* | 1 |  |  |  | 1 |  | 1 |  |  |  |  |
| *Pichia farinosa* | 1 |  | 1 |  |  |  | 1 |  |  |  |  |
| *Prototheca wickerhamii* | 1 |  |  |  | 1 |  |  |  |  | 1 |  |
| *Rhodotorula glutinis* | 1 |  |  |  | 1 |  |  |  |  | 1 |  |
| *Saccharomyces cerevisiae* | 1 | 1 |  |  |  |  | 1 |  |  |  |  |
| *Trichosporon asahii* | 1 | 1 |  |  |  |  | 1 |  |  |  |  |
| *Trichosporon mucoides* | 1 | 1 |  |  |  |  | 1 |  |  |  |  |
| **Total** | **28** | **22** | **1** | **0** | **4** | **1** | **26** | **0** | **0** | **2** | **0** |
|  |  |  |  |  |  |  |  |  |  |  |  |
| **Total number of strains (%)** | **380** | **278 (73.2)** | **47 (12.4)** | **19 (5)** | **22 (5.8)** | **14 (3.6)** | **310 (81.6)** | **33 (8.7)** | **9 (2.4)** | **18 (4.7)** | **10 (2.6)** |

No ID = No identification obtained. Mis ID = Misidentification obtained

^a^*Myroides* sp. was present in VITEK MS IVD database

^b^*Bifidobacterium* sp. was present in VITEK MS IVD database

^c^*Brevibacillus* sp. was present in VITEK MS IVD database
