# Supplementary material for: A Side by Side Comparison of Bruker Biotyper and VITEK MS: Utility of MALDI-TOF MS Technology for Microorganism Identification in a Public Health Reference Laboratory
Source: PLoS One. 2015 Dec 10;10(12):e0144878. doi: 10.1371/journal.pone.0144878 (PMC4689555; doi:10.1371/journal.pone.0144878)
Supplement: S2 Table — (DOCX) [file pone.0144878.s002.docx]

**S2 Table. Complete list of microorganism identification results when the microorganism is present only in the Bruker Biotyper database.**

| **Reference identification** | **Number of isolates** | **Bruker Biotyper** | | | | |
| --- | --- | --- | --- | --- | --- | --- |
|  |  | **Correct identification to the level of** | | |  |  |
|  |  | **Species** | **Genus** | **Complex/group** | **No ID** | **Mis ID** |
| Gram-positive cocci |  |  |  |  |  |  |
| *Enterococcus malodoratus* | 1 | 1 |  |  |  |  |
| *Enterococcus phoeniculicola* | 1 | 1 |  |  |  |  |
| *Aerococcus sanguinicola* | 1 | 1 |  |  |  |  |
| *Facklamia languida* | 1 | 1 |  |  |  |  |
| *Kocuria marina* | 1 |  | 1 |  |  |  |
| *Kocuria rhizophila* | 1 | 1 |  |  |  |  |
| *Rothia aeria* | 3 | 3 |  |  |  |  |
| *Rothia amarae* | 1 | 1 |  |  |  |  |
| *Staphylococcus condimenti* | 1 | 1 |  |  |  |  |
| *Staphylococcus fleurettii* | 1 |  | 1 |  |  |  |
| *Staphylococcus pettenkoferi* | 1 | 1 |  |  |  |  |
| *Streptococcus australis* | 2 |  |  |  |  | 2 |
| *Streptococcus infantis* | 3 |  | 2 |  |  | 1 |
| *Streptococcus pseudoporcinus* | 5 | 3 | 2 |  |  |  |
| *Vagococcus lutrae* | 1 |  | 1 |  |  |  |
| **Total** | **24** | **14** | **7** | **0** | **0** | **3** |
|  |  |  |  |  |  |  |
| Anaerobes |  |  |  |  |  |  |
| *Flavonifractor plautii* | 1 | 1 |  |  |  |  |
| **Total** | **1** | **1** | **0** | **0** | **0** | **0** |
|  |  |  |  |  |  |  |
| Other Gram-positive rods |  |  |  |  |  |  |
| *Actinobaculum schaalii* | 2 | 2 |  |  |  |  |
| *Actinobaculum urinale* | 1 |  |  |  | 1 |  |
| *Actinomyces canis* | 1 |  | 1 |  |  |  |
| *Actinomyces graevenitzii* | 2 | 2 |  |  |  |  |
| *Actinomyces naeslundii* | 2 | 1 |  |  |  | 1 |
| *Actinomyces urogenitalis* | 1 | 1 |  |  |  |  |
| *Atopobium rimae* | 1 | 1 |  |  |  |  |
| *Bacillus flexus* | 1 | 1 |  |  |  |  |
| *Bacillus halodurans* | 1 | 1 |  |  |  |  |
| *Bacillus idriensis* | 1 | 1 |  |  |  |  |
| *Bacillus nealsonii* | 1 |  |  |  | 1 |  |
| *Bacillus siralis* | 1 | 1 |  |  |  |  |
| *Corynebacterium accolens* | 1 | 1 |  |  |  |  |
| *Corynebacterium afermentans* | 1 |  | 1 |  |  |  |
| *Corynebacterium durum* | 1 | 1 |  |  |  |  |
| *Corynebacterium minutissimum* | 3 | 3 |  |  |  |  |
| *Corynebacterium riegelii* | 1 | 1 |  |  |  |  |
| *Curtobacterium* sp. | 1 |  | 1 |  |  |  |
| *Lactobacillus kalixensis* | 1 | 1 |  |  |  |  |
| *Lactobacillus reuteri* | 1 | 1 |  |  |  |  |
| *Microbacterium lacticum* | 2 |  | 1 |  |  | 1 |
| *Microbacterium oleivorans* | 1 |  | 1 |  |  |  |
| *Paenibacillus taiwanensis* | 1 |  | 1 |  |  |  |
| *Propionimicrobium lymphophilum* | 1 |  | 1 |  |  |  |
| *Sporosarcina* sp. | 1 |  |  |  | 1 |  |
| *Turicella otitidis* | 2 | 2 |  |  |  |  |
| **Total** | **33** | **21** | **7** | **0** | **3** | **2** |
|  |  |  |  |  |  |  |
| Non-fermentative Gram-negative rods |  |  |  |  |  |  |
| *Cupriavidus respiraculi* | 1 | 1 |  |  |  |  |
| *Capnocytophaga canimorsus* | 1 |  | 1 |  |  |  |
| *Burkholderia thailandensis* | 1 |  | 1 |  |  |  |
| *Burkholderia tropica* | 1 | 1 |  |  |  |  |
| *Acidovorax temperans* | 1 | 1 |  |  |  |  |
| *Cupriavidus metallidurans* | 1 | 1 |  |  |  |  |
| *Leptotrichia trevisanii* | 1 | 1 |  |  |  |  |
| *Massilia timonae* | 1 |  |  |  | 1 |  |
| *Pandoraea apista* | 1 | 1 |  |  |  |  |
| *Pandoraea sputorum* | 1 | 1 |  |  |  |  |
| *Pseudomonas pseudoalcaligenes* | 1 |  |  |  |  | 1 |
| *Roseomonas mucosa* | 2 | 2 |  |  |  |  |
| **Total** | **13** | **9** | **2** | **0** | **1** | **1** |
|  |  |  |  |  |  |  |
| Other Gram-negative bacteria |  |  |  |  |  |  |
| *Legionella anisa* | 3 | 3 |  |  |  |  |
| *Legionella bozemanae* | 2 | 2 |  |  |  |  |
| *Legionella dumoffii* | 1 | 1 |  |  |  |  |
| *Legionella jordanis* | 1 | 1 |  |  |  |  |
| *Legionella longbeachae* | 2 | 2 |  |  |  |  |
| *Legionella micdadei* | 2 | 2 |  |  |  |  |
| *Legionella rubrilucens* | 1 | 1 |  |  |  |  |
| *Neisseria sicca* | 1 |  |  |  |  | 1 |
| *Bordetella holmesii* | 1 | 1 |  |  |  |  |
| *Arcobacter butzleri* | 2 | 2 |  |  |  |  |
| *Helicobacter cinaedi* | 1 | 1 |  |  |  |  |
| *Moraxella atlantae* | 1 |  | 1 |  |  |  |
| *Neisseria weaveri* | 1 | 1 |  |  |  |  |
| *Pasteurella bettyae* | 1 | 1 |  |  |  |  |
| *Pasteurella dagmatis* | 1 | 1 |  |  |  |  |
| **Total** | **21** | **19** | **1** | **0** | **0** | **1** |
|  |  |  |  |  |  |  |
| Mycobacteriaceae |  |  |  |  |  |  |
| *Mycobacterium abscessus* | 4 |  | 3 |  | 1 |  |
| *Mycobacterium bovis*^a^ | 3 |  | 1 | 2 |  |  |
| *Mycobacterium branderi* | 2 |  | 2 |  |  |  |
| *Mycobacterium celatum* | 1 | 1 |  |  |  |  |
| *Mycobacterium chelonae* | 4 |  | 3 |  | 1 |  |
| *Mycobacterium conceptionense* | 2 |  | 1 |  | 1 |  |
| *Mycobacterium florentinum* | 1 | 1 |  |  |  |  |
| *Mycobacterium gastri* | 1 |  | 1 |  |  |  |
| *Mycobacterium gordonae* | 4 |  | 4 |  |  |  |
| *Mycobacterium interjectum* | 2 | 1 | 1 |  |  |  |
| *Mycobacterium kumamotonense* | 2 | 2 |  |  |  |  |
| *Mycobacterium marinum* | 3 | 2 | 1 |  |  |  |
| *Mycobacterium peregrinum* | 2 | 2 |  |  |  |  |
| *Mycobacterium simiae* | 2 | 1 |  |  | 1 |  |
| *Mycobacterium* sp.^a^ | 9 |  | 3 |  | 6 |  |
| *Mycobacterium szulgai* | 2 |  | 2 |  |  |  |
| *Mycobacterium tuberculosis*^a^ | 2 |  | 2 |  |  |  |
| *Mycobacterium xenopi* | 1 |  | 1 |  |  |  |
| **Total** | **47** | **10** | **25** | **2** | **10** | **0** |
|  |  |  |  |  |  |  |
| Actinomycetes |  |  |  |  |  |  |
| *Gordonia aichiensis* | 1 |  | 1 |  |  |  |
| *Gordonia sputi* | 2 | 1 | 1 |  |  |  |
| *Nocardia abscessus* | 2 |  |  |  | 2 |  |
| *Nocardia cyriacigeorgica* | 2 |  |  |  | 2 |  |
| *Nocardia farcinica* | 3 |  |  |  | 3 |  |
| *Nocardia nova* | 3 | 1 | 1 |  | 1 |  |
| *Nocardia otitidiscaviarum* | 1 |  |  |  | 1 |  |
| *Nocardia thailandica* | 1 |  |  |  | 1 |  |
| *Nocardia veterana* | 1 |  | 1 |  |  |  |
| *Nocardiopsis alba* | 1 |  | 1 |  |  |  |
| *Streptomyces* sp. | 3 |  | 1 |  | 2 |  |
| **Total** | **20** | **2** | **6** | **0** | **12** | **0** |
|  |  |  |  |  |  |  |
| Filamentous fungi |  |  |  |  |  |  |
| *Arthrographis kalrae* | 1 | 1 |  |  |  |  |
| *Aspergillus glaucus* | 1 | 1 |  |  |  |  |
| *Aspergillus terreus* | 1 | 1 |  |  |  |  |
| *Aureobasidium pullulans* | 1 |  | 1 |  |  |  |
| *Chaetomium globosum* | 1 | 1 |  |  |  |  |
| *Chrysosporium* sp. | 2 |  | 1 |  | 1 |  |
| *Epidermophyton floccosum* | 1 |  |  |  | 1 |  |
| *Exophiala dermatitidis* | 1 |  |  |  | 1 |  |
| *Lichtheimia corymbifera* | 1 |  | 1 |  |  |  |
| *Microsporum canis* | 1 | 1 |  |  |  |  |
| *Microsporum gypseum* | 1 |  |  |  | 1 |  |
| *Microsporum persicolor* | 1 |  | 1 |  |  |  |
| *Mucor* sp. | 1 | 1 |  |  |  |  |
| *Phoma* sp. | 1 |  |  |  | 1 |  |
| *Pseudallescheria boydii* | 1 | 1 |  |  |  |  |
| *Rhizomucor pusillus* | 1 | 1 |  |  |  |  |
| *Rhizopus stolonifer* | 1 | 1 |  |  |  |  |
| *Scedosporium apiospermum* | 2 | 2 |  |  |  |  |
| *Scedosporium prolificans* | 1 | 1 |  |  |  |  |
| *Scopulariopsis brevicaulis* | 1 |  |  |  | 1 |  |
| *Trichophyton tonsurans* | 1 |  |  |  | 1 |  |
| *Trichophyton interdigitale* | 1 |  | 1 |  |  |  |
| *Trichophyton rubrum* | 2 | 1 |  |  | 1 |  |
| **Total** | **26** | **13** | **5** | **0** | **8** | **0** |
|  |  |  |  |  |  |  |
| Yeast |  |  |  |  |  |  |
| *Candida nivariensis* | 1 | 1 |  |  |  |  |
| *Candida orthopsilosis* | 1 | 1 |  |  |  |  |
| *Candida pararugosa* | 1 | 1 |  |  |  |  |
| *Candida palmioleophila* | 1 | 1 |  |  |  |  |
| *Cryptococcus gattii* | 1 |  | 1 |  |  |  |
| *Pseudozyma* sp. | 1 |  | 1 |  |  |  |
| *Trichosporon cutaneum* | 1 |  |  |  | 1 |  |
| **Total** | **7** | **4** | **2** | **0** | **1** | **0** |
|  |  |  |  |  |  |  |
| **Total number of strains (%)** | **192** | **93 (48.4)** | **55 (28.7)** | **2 (1)** | **35 (18.2)** | **7 (3.6)** |

No ID = No identification obtained. Mis ID = Misidentification obtained

^a^For these *Mycobacteria*, they were referenced in the VITEK MS IVD database. However, identification were not performed with VITEK MS since the viability test failed.
