# Supplementary material for: A Side by Side Comparison of Bruker Biotyper and VITEK MS: Utility of MALDI-TOF MS Technology for Microorganism Identification in a Public Health Reference Laboratory
Source: PLoS One. 2015 Dec 10;10(12):e0144878. doi: 10.1371/journal.pone.0144878 (PMC4689555; doi:10.1371/journal.pone.0144878)
Supplement: S3 Table — (DOCX) [file pone.0144878.s003.docx]

**S3 Table. Complete list of microorganism identification results when the microorganism is present only in the VITEK MS IVD database.**

| **Reference identification** | **Number of isolates** | **VITEK MS (IVD)** | | | | |
| --- | --- | --- | --- | --- | --- | --- |
|  |  | **Correct identification to the level of** | | |  |  |
|  |  | **Species** | **Genus** | **Complex/group** | **No ID** | **Mis ID** |
| Gram-positive cocci |  |  |  |  |  |  |
| *Arthrobacter soli* | 1 |  |  |  | 1 |  |
|  |  |  |  |  |  |  |
| Other Gram-positive rods |  |  |  |  |  |  |
| *Microbacterium paraoxydans* | 2 | 2 |  |  |  |  |
|  |  |  |  |  |  |  |
| Non- fermentative Gram-negative rods |  |  |  |  |  |  |
| *Vibrio cholerae* | 2 | 2 |  |  |  |  |
|  |  |  |  |  |  |  |
| Other Gram-negative bacteria |  |  |  |  |  |  |
| *Haemophilus haemolyticus* | 1 | 1 |  |  |  |  |
|  |  |  |  |  |  |  |
| Mycobacteriaceae |  |  |  |  |  |  |
| *Mycobacterium scrofulaceum* | 1 | 1 |  |  |  |  |
|  |  |  |  |  |  |  |
| Filamentous fungi |  |  |  |  |  |  |
| *Aspergillus calidoustus* | 1 |  |  | 1 |  |  |
| *Aspergillus sydowii* | 1 | 1 |  |  |  |  |
| *Penicillium purpurogenum* | 1 |  |  |  | 1 |  |
|  |  |  |  |  |  |  |
| Yeast |  |  |  |  |  |  |
| *Cryptococcus terreus* | 1 | 1 |  |  |  |  |
| *Cryptococcus albidus* | 1 | 1 |  |  |  |  |
|  |  |  |  |  |  |  |
| **Total number of strains (%)** | **12** | **9 (75)** | **0** | **1 (8.3)** | **2 (16.7)** | **0** |

No ID = No identification obtained. Mis ID = Misidentification obtained
